# Hepatoprotective Activity of *Nelumbo nucifera* Gaertn. Seedpod Extract Attenuated Acetaminophen-Induced Hepatotoxicity

**DOI:** 10.3390/molecules27134030

**Published:** 2022-06-23

**Authors:** Hui-Hsuan Lin, Jen-Ying Hsu, Chiao-Yun Tseng, Xiao-Yin Huang, Hsien-Chun Tseng, Jing-Hsien Chen

**Affiliations:** 1 Department of Medical Laboratory and Biotechnology, Chung Shan Medical University, Taichung City 40201, Taiwan; linhh@csmu.edu.tw; 2 Department of Nutrition, Chung Shan Medical University, Taichung City 40201, Taiwan; jyhsu0530@gmail.com (J.-Y.H.); winnielovejb3131@gmail.com (C.-Y.T.); yin17@hotmail.com.tw (X.-Y.H.); 3 Department of Radiation Oncology, Chung Shan Medical University Hospital, Taichung City 40201, Taiwan; 4 Department of Radiation Oncology, School of Medicine, Chung Shan Medical University, Taichung City 40201, Taiwan

**Keywords:** acetaminophen, lotus seedpod extract, hepatotoxicity, apoptosis, inflammation

## Abstract

The aim is to investigate the effect of lotus (*Nelumbo nucifera* Gaertn.) seedpod extract (LSE) on acetaminophen (APAP)-induced hepatotoxicity. LSE is rich in polyphenols and has potent antioxidant capacity. APAP is a commonly used analgesic, while APAP overdose is the main reason for drug toxicity in the liver. Until now, there has been no in vitro test of LSE in drug-induced hepatotoxicity responses. LSEs were used to evaluate the effect on APAP-induced cytotoxicity, ROS level, apoptotic rate, and molecule mechanisms. The co-treatment of APAP and LSEs elevated the survival rate and decreased intracellular ROS levels on HepG2 cells. LSEs treatment could significantly reduce APAP-induced HepG2 apoptosis assessed by DAPI and Annexin V/PI. The further molecule mechanisms indicated that LSEs decreased Fas/FasL binding and reduced Bax and tBid to restore mitochondrial structure and subsequently suppress downstream apoptosis cascade activation. These declines in COX-2, NF-κB, and iNOS levels were observed in co-treatment APAP and LSEs, which indicated that LSEs could ameliorate APAP-induced inflammation. LSE protected APAP-induced apoptosis by preventing extrinsic, intrinsic, and JNK-mediated pathways. In addition, the restoration of mitochondria and inflammatory suppression in LSEs treatments indicated that LSEs could decrease oxidative stress induced by toxic APAP. Therefore, LSE could be a novel therapeutic option for an antidote against overdose of APAP.

## 1. Introduction

Acetaminophen (APAP) is an analgesic and antipyretic agent which is broadly utilized in either prescription or over-the-counter products. The maximum daily dose of APAP is 4 g/day for an adult. Under the therapeutic dose, APAP is an effective and safe drug for acute or chronic pain control which is recommended by the WHO as the first-option antipyretic [1]. In chronic conditions such as cancer pain and osteoarthritis, patients required long-term high dose use of APAP [2], at the same time, increasing the risk of APAP poisoning. Clinical studies indicated that high dose use of APAP for controlling chronic pain caused toxic effects in many organs as well as increased long-term mortality [3,4]. The major APAP metabolism organ is liver. Its poisoning resulted from depletion of glutathione and toxic metabolites accumulation [5]. Qingyun Bai et al. revealed that long-term high dose APAP administration in mice led to liver fibrosis [6]. N-acetyl cysteine (NAC) is currently the only FDA-approved antidote for the treatment of APAP poisoning. The study of antidotes to protect from high-dose use of APAP hepatotoxicity is limited. It is necessary to provide a novo antidote to protect against the liver damage caused by long-term high dose APAP exposure.

Lotus (*Nelumbo nucifera* Gaertn.), also called Chinese water lily, is a perennial aquatic plants. Lotus is mainly grown in China, Japan, and Singapore. All parts of the lotus, including the seed, flower, and root, possess bioactivity and are also known as Chinese traditional medicines. Numerous phenolic compounds and alkaloids have been identified in lotus [7,8]. Lotus seedpod is the byproduct of lotus and is often discarded during the processing of Chinese medicine. So far, the extract of lotus seedpod is the only part rich in proanthocyanidins [9]. Previous studies reported that proanthocyanidins possessed anti-tumor effects [10], potent antioxidant capacity [11,12], inhibited advanced glycation-end products formation [9], neuroprotective effects [13], restrained the inflammatory response [14], and attenuated age-related deficits in cognitive functions [15]. In the latest study, the extract of lotus seedpod was investigated to protect LPS-induced hepatotoxicity through inhibiting pro-inflammatory cytokines mediators expression [16], and reduced lipid accumulation and lipotoxicity in hepatocytes [17]. However, the effect of LSE on drug-induced hepatotoxicity is still unclear.

APAP is a commonly used analgesic; the study indicated that APAP overdose was the main reason for drug toxicity [18]. The study aimed to investigate the protective effects of LSE and its possible mechanisms in APAP overdose-induced hepatotoxicity. Until now, there has been no in vitro test for this aspect of drug-induced hepatotoxicity responses. The present study designed an in vitro model using HepG2 cells and treated them with a toxic concentration of APAP in the presence or absence of LSE or its major composition epigallocatechin (EGC) [16] for exploring and comparing the protective effects and mechanisms.

## 2. Results

### 2.1. LSE Prevented Toxic APAP-Induced Cell Death in HepG2 Cells

**Figure 1:**
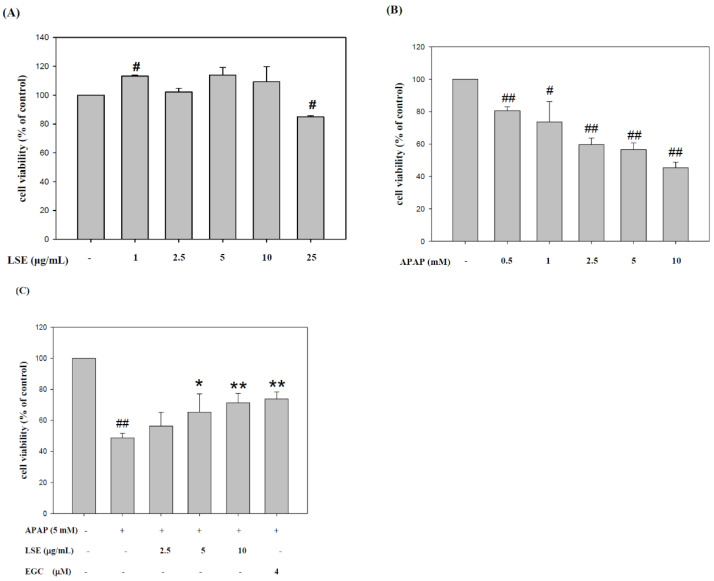
LSE or EGC co-treated with toxic dose of APAP elevated HepG2 cell survival rate. First, the cytotoxic effects of (**A**) LSE and (**B**) APAP in various concentrations on HepG2 cells were evaluated. (**C**) The HepG2 cell viability of APAP in 5 mM with/without LSE in 2.5, 5, and 10 µg/mL or 4 µM EGC. All data are presented as means ± SD of three independent experiments. # *p* < 0.05, ## *p* < 0.01 compared with the control group. * *p* < 0.05, ** *p* < 0.01 compared with the APAP group.

First, various concentrations of LSE and APAP were tested in HepG2 cells. The cytotoxicity of LSE in various concentrations on HepG2 showed that there were no cytotoxic effects on HepG2 cells in LSE 2.5, 5, and 10 μg/mL (Figure 1A). The cell survival rates of APAP in different concentrations were analyzed and found that APAP was toxic in over 2.5 mM in HepG2 cells (Figure 1B). According to the results, 2.5, 5, and 10 μg/mL LSE and 5 mM APAP were selected for further study. To determine the cytoprotective effect of LSE in toxic APAP-induced cell death, LSE in 2.5, 5, and 10 μg/mL were co-treated with 5 mM APAP for 24 h. As shown in Figure 1C, the cell survival rates were significantly elevated by 8–25% with increasing LSE concentrations. The calculation and concentration scale were replaced for EGC, with the result that EGC of 4 microM is equal to LSE of 10 μg/mL, EGC changed the same unit by approximately 1.22 μg/mL. In addition, the increase of HepG2 survival in 10 μg/mL LSE treatment was similar to 4 μM EGC treatment. These results indicate that the intervention of LSE could reduce toxic APAP-induced cell death.

### 2.2. LSE Reduced Intracellular ROS Level and Apoptosis Rate in Toxic APAP Condition

**Figure 2:**
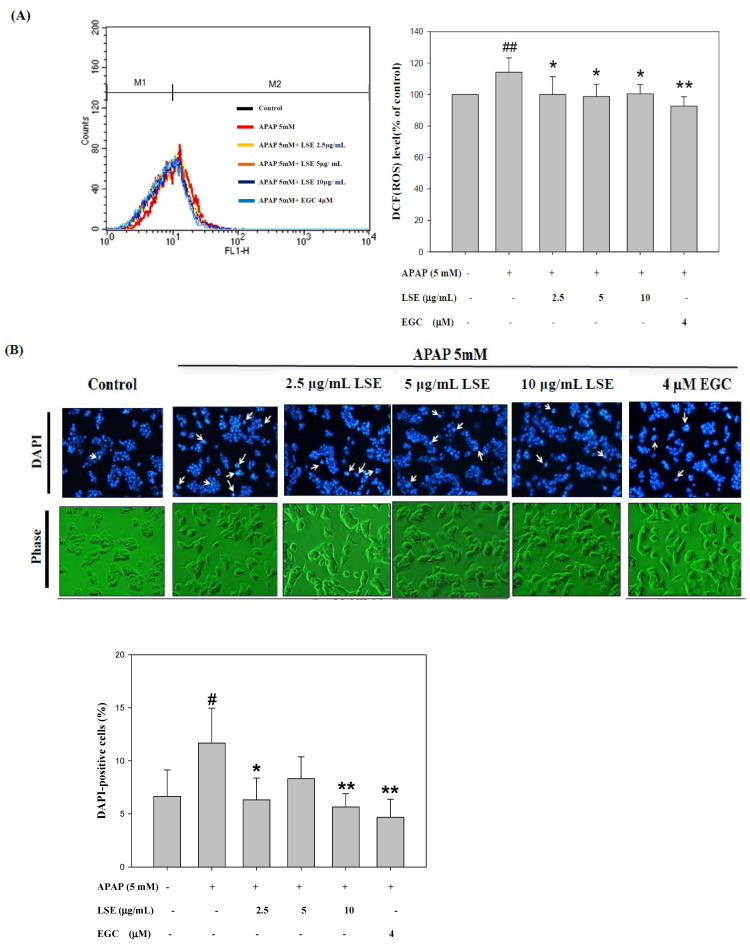

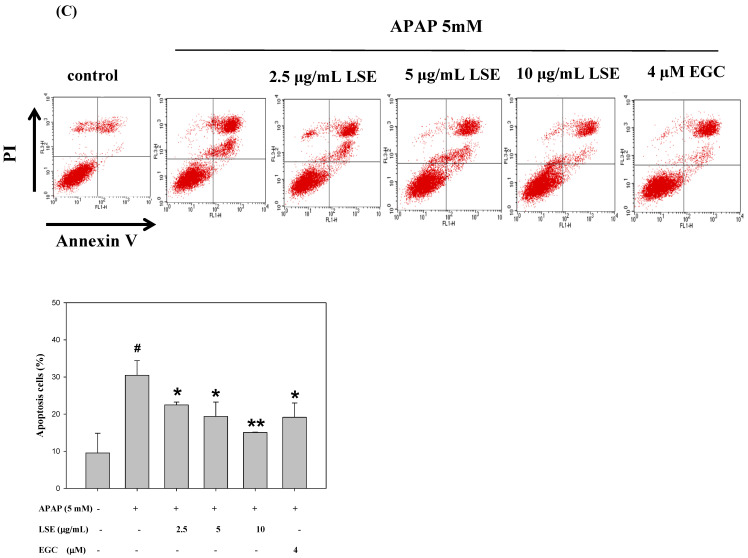
Effect of APAP with/without LSE (2.5, 5, and 10 µg/mL) or EGC (4 µM) on (**A**) intracellular ROS levels and (**B**) the cellular morphology was stained by DAPI. Apoptotic nuclei are indicated by arrows. Apoptotic values are presented as percentage of apoptotic cells divided by total number of cells. (**C**) The apoptosis rate of HepG2 cells analyzed by flow cytometry with annexin V/PI double staining after treating LSE or EGC with or without APAP for 24 h. Upper right zone indicates the late stage of apoptotic cells (Annexin V and PI positive). All data were presented as means ± SD of three independent experiments. # *p* < 0.05, ## *p* < 0.01 compared with the control group. * *p* < 0.05, ** *p* < 0.01 compared with the APAP group.

The intracellular ROS levels in toxic APAP with/without LSE and EGC were determined and the results are shown in Figure 2A. The ROS level in the APAP group was significantly higher by 14% than in the control group. With co-treatment of LSE with APAP, the ROS level significantly decreased by 12%, 14%, and 12% compared with the APAP group and was similar to those which in the control group and the EGC treatment group. Apoptosis morphological changes were stained by DAPI. As shown in Figure 2B, the fluorescence intensity in toxic APAP treatment was significantly increased compared to the control group, which indicated that the nucleus condensation in HepG2 cells occurred when exposed to toxic APAP. However, the phenomena were attenuated in co-treatment with LSE or EGC since the fluorescence intensities were significantly reduced compared with APAP group (Figure 2B). The apoptosis rates in different treatments were stained by Annexin V/PI and detected by flow cytometry. The results were shown in Figure 2C and the upper right zone indicated the late stage of apoptotic cells. The apoptosis rate in APAP group was significantly increased by 21% compared with the control group, but co-treatment of LSE or EGC reduced apoptotic cells compared with the APAP group (Figure 2C). These results show that co-treatment of LSE with toxic APAP prevented intracellular ROS levels as well as APAP-induced cell apoptosis.

### 2.3. LSE Inhibited APAP-Induced Apoptosis in Extrinsic and Intrinsic Pathways

**Figure 3:**
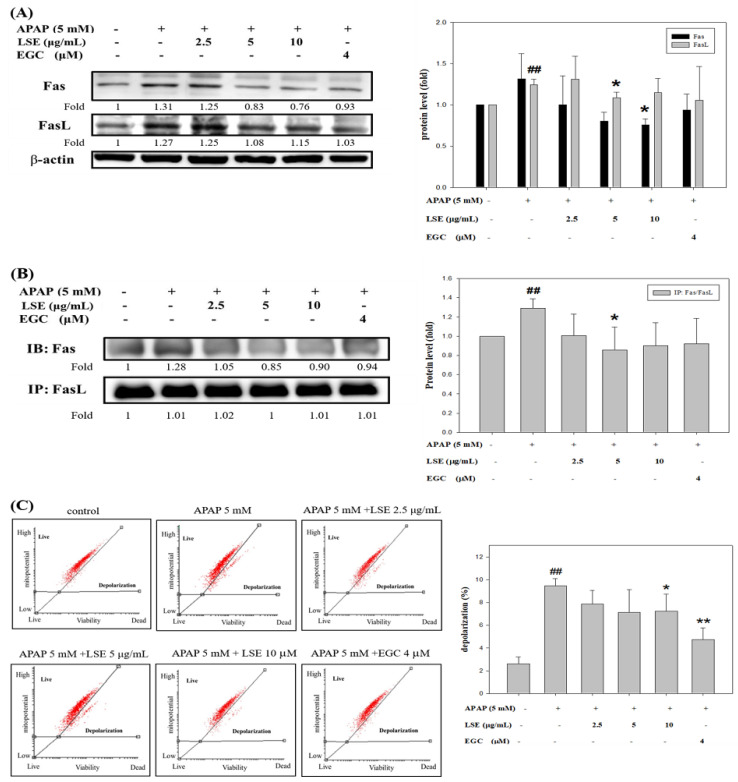

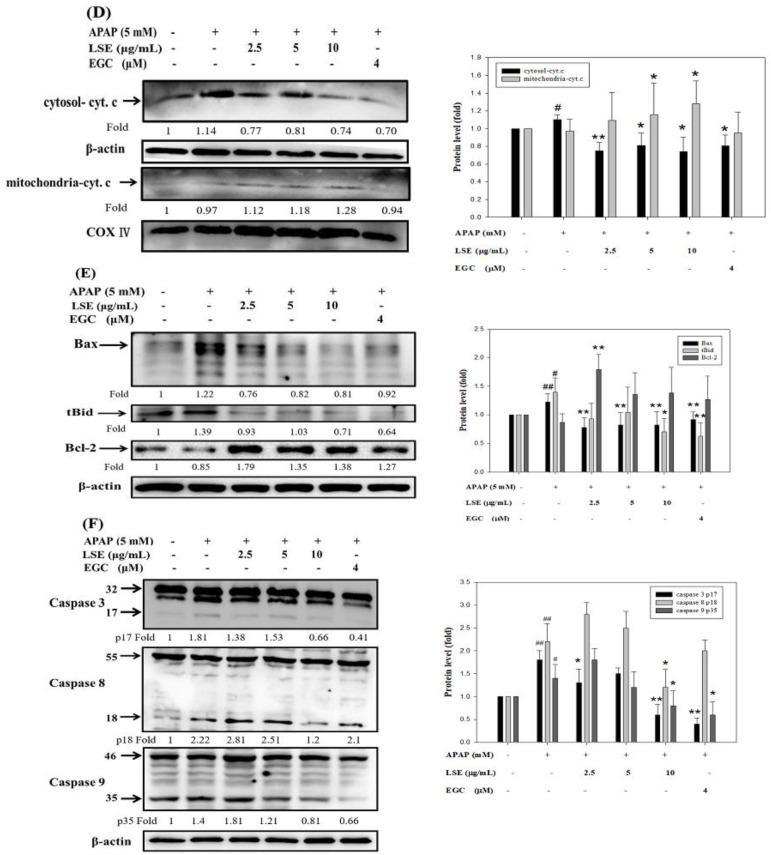
Effect of LSE on extrinsic and intrinsic apoptotic pathways in toxic APAP-treated HepG2 cells. HepG2 cells were treated with APAP (5 mM) in the presence or absence of LSE (2.5, 5, 10 µg/mL) or EGC (4 µM) for 24 h. (**A**) The protein levels of Fas and FasL were analyzed by Western blotting. (**B**) The protein levels of Fas and FasL were analyzed by IP. The cell extracts were IP with FasL. The precipitated complex was examined for immunoblotting (IB) using Fas antibody. (**C**) The mitochondrial membrane depolarization was assayed by JC-1staining with flow cytometry. Percentage of mitochondrial depolarization is plotted for various treatment groups. (**D**) The protein levels of cytosol and mitochondria cytochrome c were analyzed by Western blotting. (**E**) The protein levels of Bax, tBid, and Bcl-2 were analyzed by Western blotting. (**F**) The protein levels of caspase 3, 8, and 9 of each group were analyzed by Western blotting. β-actin and COX 4 were served as an internal control of cell cytosol and mitochondria. All data were presented as means ± SD of three independent experiments. # *p* < 0.05, ## *p* < 0.01 compared with the control group. * *p* < 0.05, ** *p* < 0.01 compared with the APAP group.

Based on the results mentioned above, the effect of LSE on further apoptosis-related mechanisms was investigated. To explore whether the death receptor, Fas ligand (FasL) and Fas receptor (Fas), was involved in APAP-induced liver injury, the change of protein levels and interaction of FasL and Fas were analyzed. The results revealed that Fas receptor and FasL expressions were increased in the APAP group when compared with the control group, but both were decreased with co-treatment with LSE (Figure 3A).

Further, the immunoprecipitation assay verified that co-treatment of LSE and EGC could reduce Fas/FasL complex formation, especially in LSE 5 μg/mL (Figure 3B). Next, the mitochondrial depolarization was measured and found which significantly increased by 262% in the APAP group compared with the control group (Figure 3C). Co-treatment of APAP with LSE or EGC decreased mitochondrial depolarization, especially in LSE 10 μg/mL and EGC 4 μM, which significantly reduced 24% and 50%, respectively, compared with the APAP group (Figure 3C). A previous study reported that mitochondrial depolarization was related to cytochrome c release [19]. Cytochrome c level in mitochondria was reduced whereas it was increased in cytosol in toxic APAP treatment (Figure 3D). However, the co-treatment of APAP with LSE revealed that the cytochrome c was retained in mitochondria (Figure 3D). Anti-apoptosis protein Bcl-2 and pro-apoptosis protein levels of Bax and tBid were analyzed and presented in Figure 3E. In the toxic APAP concentration group, Bax and tBid levels were increased but Bcl-2 level was decreased when compared with the control group (Figure 3E), while in LSE and APAP co-treatment groups, both Bax and tBid levels were significantly reduced (Figure 3E), especially in 10 μg/mL LSE treatment, which were similar to the EGC group. Bcl-2 levels in co-treatment LSE and APAP group were increased by 107%, 56%, and 60% compared with the APAP group (Figure 3E). The active forms of caspase 3, 8, and 9 were analyzed in each group. These three protein levels in APAP group were significantly higher than the control group but reduced in 10 μg/mL LSE group and similar to the results in the EGC treatment group (Figure 3F).

According to these results, toxic APAP-induced apoptosis was related to both extrinsic and intrinsic pathways. However, the co-treatment of APAP with LSE could not only interrupt the interaction of FasL and Fas but retain mitochondrial integrity to prevent the downstream apoptotic cascade activation.

### 2.4. LSE Attenuated ASK 1/MEK-7/JNK Mediated Apoptosis Signal Induced by Toxic APAP

**Figure 4:**
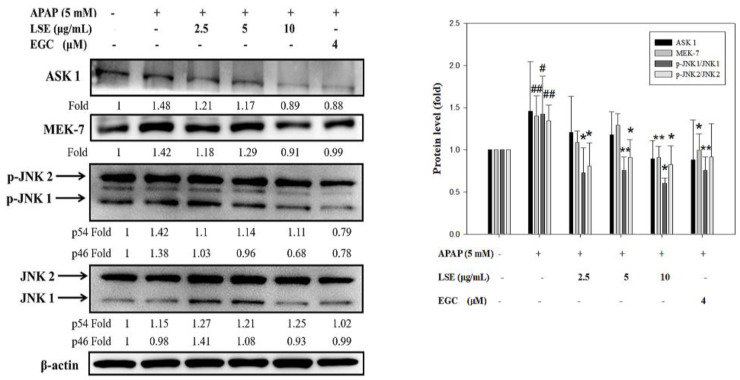
Effect of LSE and EGC on ASK 1/MEK-7/JNK signaling pathways in toxic APAP-treated HepG2 cells. HepG2 cells were treated with APAP (5 mM) in the presence or absence of LSE (2.5, 5, 10 µg/mL) or EGC (4 µM) for 24 h. The protein levels of ASK 1, MEK7, JNKs, and p-JNKs were analyzed by Western blotting. β-actin served as an internal control. The quantitative data were presented as means ± SD of three independent experiments. # *p* < 0.05, ## *p* < 0.01 compared with the control group. * *p* < 0.05, ** *p* < 0.01 compared with the APAP group.

The extensive oxidative stress and death receptor signal transduction activated ASK1 and MEK 7 which regulate JNK phosphorylation [20,21]. In the present study, ASK1, MEK7, and the ratio of p-JNK1/JNK1 and p-JNK2/JNK2 expressions were significantly increased in APAP group compared with control group (Figure 4). In LSE and APAP co-treatment groups, ASK1 and MEK7 levels were lower by 8–39% than the APAP group. In addition, the ratio of p-JNK1/JNK1 and p-JNK2/JNK2 were significantly reduced by 32–58% in LSE groups compared with APAP group (Figure 4). Similar results were shown in the EGC group. These results indicate that LSE intervention inhibited ASK 1/MEK-7/JNK-mediated apoptosis.

### 2.5. LSE Suppressed Toxic APAP-Induced Inflammation by Reducing Inflammatory Factors Level

**Figure 5:**
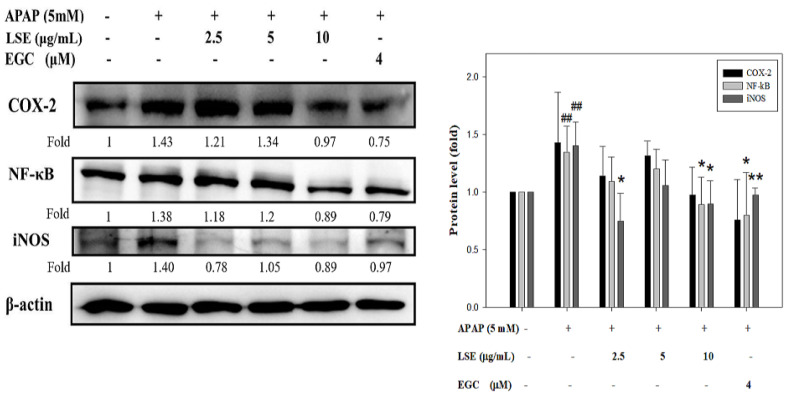
Effect of LSE and EGC on inflammatory factors in toxic APAP-treated HepG2 cells. HepG2 cells were treated with APAP (5 mM) in the presence or absence of LSE (2.5, 5, 10 µg/mL) or EGC (4 µM) for 24 h. The protein levels of COX-2, NF-κB, and iNOS were analyzed by Western blotting. β-actin served as an internal control. The quantitative data are presented as means ± SD of three independent experiments. ## *p* < 0.01 compared with the control group. * *p* < 0.05, ** *p* < 0.01 compared with the APAP group.

The changes of NF-κB, COX-2, and iNOS were determined to explore the effect of LSE on toxic APAP-induced inflammation. As shown in Figure 5, NF-κB, COX-2, and iNOS protein levels in APAP group were significantly higher than in the control group, while in LSE groups, NF-κB, COX-2, and iNOS protein levels were decreased when compared with the APAP group, especially in LSE 10 μg/mL group which was similar to EGC groups.

## 3. Discussion

The FDA has been concerned about APAP-toxicity and has subjected it to Advisory Committees. APAP-induced hepatotoxicity is caused by its reactive metabolite N-acetyl-p-benzoquinone imine (NAPQI) which is irreversibly quenched by the glutathione-SH group as a non-toxic metabolite and excreted into urine [22]. Unfortunately, an overdose of APAP caused considerable NAPQI formation and consequently depleted GSH and covalently bonded to the SH group in cellular or mitochondrial protein to form protein-adducts leading to mitochondrial dysfunction [5,23,24]. APAP is usually considered a common drug; this painkiller can lead to liver injury after overdoses [25]. EGC was confirmed as the main component of LSE [16]. A recent study identified that EGC from green tea protects against APAP-induced liver injury [26] leading to the antioxidant capacity of EGC. In addition, overdose of APAP exposure triggered the apoptosis pathway and inflammatory reactions [27,28]. The present study investigated the effects of LSE on APAP overdose-induced liver injury through in vitro study. The results revealed that overdose of APAP caused severe hepatotoxicity which was involved in apoptosis, oxidative stress, and inflammatory response leading to cell death. LSE treatment could prevent toxic APAP-induced apoptosis and inflammation.

The present study revealed that LSE reduced cell apoptosis rate which was involved with both extrinsic and intrinsic pathways. The extrinsic apoptosis pathway is initiated by death receptors, including TNF and Fas. The binding of Fas/FasL recruited caspase 8 to induce a downstream factor of caspase 3 activity and resulting in cell death [29]. Another signaling of caspase 8 induced Bid cleaving to tBid and cooperating with Bax. Both tBid and Bax were translocated to mitochondria and lead to mitochondrial permeability [28]. This process was crosslinked with an intrinsic apoptosis pathway. The intrinsic pathway was triggered by intracellular stimuli and consequently repressed anti-apoptosis proteins, Bcl-2, coupled with enhanced pro-apoptosis proteins Bax and Bid [30]. The present study revealed that LSE in high concentrations inhibited not only Fas/FasL complex formation but also reduced Bid cleavage and ceased the sequential activation. Mitochondrial membrane depolarization and the expression of cytochrome c were increased in toxic APAP exposure. Mitochondrial membrane depolarization-induced membrane permeability resulted in cytochrome c and caspase 9, which reside in mitochondria, being released into the cytoplasm [30,31]. However, LSE treatment repressed mitochondrial depolarization to restore cytochrome c residing in mitochondria. In addition, active caspase 3 and 9 levels decreased in LSE treatment groups which indicated that LSE treatment could prevent toxic dose APAP-induced apoptosis. These results demonstrated that LSE treatment could rescue HepG2 cells from toxic APAP treatment by inhibiting extrinsic and intrinsic apoptosis pathways.

Overwhelmed toxic metabolite NAPQI covalently bound to mitochondrial proteins and led to mitochondrial dysfunction following reactive oxide species generation, oxidative stress, and causing mitochondrial permeability transition [32,33]. Moreover, death receptor signaling transduction and inflammatory responses were sources of intracellular ROS [34]. Sustained oxidative stress and ROS production activated ASK1 and MEK7, one of the MAPK family proteins, and subsequently phosphorylated JNK to translocate to mitochondria [35]. Activated JNK led mitochondria to produce more ROS generation and the self-amplified JNK activation pathway exacerbated mitochondrial permeability transition [36]. The deleterious cycle and apoptotic stimuli from death receptor-induced ASK 1 activity and JNK phosphorylation impair mitochondrial function and promote pro-apoptotic proteins, including Bax, cytochrome c, and caspase 9 activation [21,37]. As agreed with previous studies, the present study observed that mitochondrial membrane depolarization and ROS formation were increased in the toxic concentration of APAP, which probably indicated mitochondrial permeability transition. However, LSE treatment reduced mitochondrial depolarization and ROS production. Moreover, the expressions of ASK1, MEK7, and the ratio of p-JNK1/JNK1 and p-JNK2/JNK2 were decreased especially in high LSE concentrations. We could demonstrate that LSE could act against toxic dose APAP-induced oxidative stress and apoptosis by repressing the ASK 1/MEK7/JNK signaling pathway.

Inflammation-mediated liver damages played a pivotal role in APAP overdose-induced hepatotoxicity which is involved with NF-κB activation and certain cytokines secretion, such as TNF-α and IL-1β [38]. NF-κB is a transcription factor that was activated by oxidative stress or inflammatory cytokines and then translocated into the nucleus to induce chemokines and pro-inflammatory enzyme expressions, including iNOS and COX-2 [39,40]. Previous studies reported that APAP overdose-induced inflammatory reaction and oxidative stress increased iNOS expression and reactive nitrogen species (RNS) production which further formed peroxynitrite [34,41]. The character of COX-2 is similar to iNOS, both of which induced their activity for inflammatory response. The previous study revealed that the transgenic mice lock COX-2 became more susceptible to APAP-induced liver injuries [42]. The present study found toxic APAP-induced NF-κB, iNOS, and COX-2 expressions in HepG2 cells. However, the co-treatment of APAP with 10 μg/mL LSE group reduced these factors’ expressions. The results indicated that the LSE intervention ameliorated toxic APAP-induced inflammatory by suppressing the activation and translocation of NF-κB, subsequently ceasing the NF-κB-dependent enzymes, COX-2, and iNOS expressions.

The strengths of our study give the original viewpoint of these findings that may open interesting perspectives to the strategy for treatment of APAP-induced liver injury. However, the limitations are that the study has not been investigated in vivo and clinical research has not been done, which are being considered for future studies.

## 4. Materials and Methods

### 4.1. Preparation of Lotus Seedpod Extracts (LSE)

**Figure 6:**
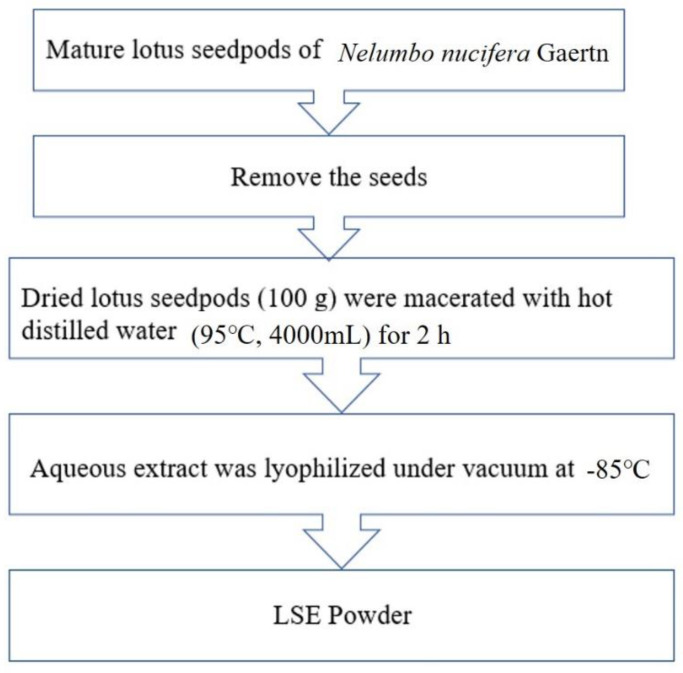
LSE preparation process. The dried lotus seedpods (*Nelumbo nucifera* Gaertn.) (100 g) were macerated with hot distilled water (95 °C, 4000 mL) for 2 h. The decoction was filtered and lyophilized as lotus seedpods extract powder (LSE).

The lotus seedpods (*Nelumbo nucifera* Gaertn.) were obtained from Tainan, Taiwan. Seeds were removed from the lotus seedpods before extraction. The dried lotus seedpods (100 g) were macerated with hot distilled water (95 °C, 4000 mL) for 2 h. After the solid lotus seedpod was filtered, the aqueous extract was lyophilized under vacuum at −85 °C as the lotus seedpods extracts powder (LSE). The yield was approximately 17.2% of dried materials. The functional components were described in the previous study. The components of LSE were determined by HPLC analysis and the Folin–Ciocalteau method. EGC (12.5 ± 1.2%) was identified to be presented at the highest level in LSE [16]. The preparation process figure of LSE is shown in Figure 6.

### 4.2. Cell Line and Treatment

The human hepatocellular carcinoma cell line HepG2 was purchased from the Bioresource Collection and Research Center (BCRC, Food Industry Research and Development Institute, Hsinchu, Taiwan, ROC). HepG2 cells were cultured in MEM medium supplemented with 2 mM L-glutamine, 1.5 g/L sodium bicarbonate, 0.1 mM non-essential amino acids, 1.0 mM sodium pyruvate, 100 U/mL penicillin, 100 μg/mL streptomycin, and 10% fetal bovine serum (FBS) at 37 °C in a humidified atmosphere of 5% CO_2_ [43]. The HepG2 cells were seeded (5 × 10^5^ cells/mL in MEM) in 6-well plates before treatment.

### 4.3. Cell Viability by Trypan Blue Assay

As described previously [44], to determine the cytotoxicity of cell survival, the trypan blue exclusion assay was performed. Cells were pre-treated with LSE (2.5, 5, and 10 μg/mL) or EGC (4 μM) for 1 h and subsequently treated with APAP (5 mM) for 24 h. The number of cells stained with trypan blue and the live cells were counted to determine cell survival rate.

### 4.4. DAPI Stain Assay

HepG2 cells were seeded in $4.5\times 10^{5}$ cells/well and cultured in 6-well plates. To the cells were added LSE (2.5, 5, and 10 μg/mL) or EGC (4 μM) before APAP (5 mM). After 24 h, the medium was removed and rinsed with warm PBS followed by 4% paraformaldehyde fixation for 30 min. Next, DAPI (Sigma-Aldrich, St Louis, MO, USA) was diluted with PBS and the nucleus stained for 30 min at room temperature in the dark. Cell morphology was pictured by fluorescence microscopy and quantified by ImageJ software [45].

### 4.5. Annexin V/Propidium Iodide (PI) Stain Assay

The steps of Annexin V/PI stain assay mainly referred to FITC Annexin V Apoptosis Detection Kit (#556547, BD Biosciences, San Jose, CA, USA) [46]. HepG2 cells were harvested, centrifuged, and resuspended with the 1X binding buffer of the kit. Then, 100 μL cells suspension was transferred to 1.5 mL Eppendorf and stained with 5 μL annexin V and 5 μL PI at room temperature for 15 min in a dark room and then added 400 μL 1× binding buffer. Cell apoptosis was analyzed by FACS101 flow cytometer (BD Biosciences, Franklin Lakes, NJ, USA).

### 4.6. JC-1 Assay for Mitochondrial Membrane Depolarization Analysis

JC-1 stain was a cationic dye that accumulated in mitochondrial membrane based on membrane potential [17]. Briefly, the medium was removed and HepG2 cells were washed with PBS after treatment. JC-1 stain was prepared in medium (0.25 µg/mL) and added 1 mL to each well for 15 min. After incubation, cells were washed with PBC and evaluated by fluorescence microscopy.

### 4.7. DCFH-DA Assay for ROS Analysis

A chemically reduced form of fluorescein, 2′,7′-dichlorodihydrofluorescein diacetate (DCFH-DA), was used as an indicator to quantify reactive oxygen species (ROS) in cells. 2′,7′-dichlorofluorescein diacetate (DCF-DA) stain was diluted with medium and added, firstly incubating for 1 h [47]. After removing the DCF-DA stain, APAP (5 mM) with/without LSE (2.5, 5, and 10 μg/mL) or EGC (4 μM) were added to HepG2 cells and incubated for 24 h. Following the treatment, cells were harvested and suspended with PBS and counted using FACS101 flow cytometer.

### 4.8. Mitochondria Isolation

Mitochondria from HepG2 cells were isolated using the Mitochondrial Isolation Kit from Thermo (Rockford, IL, USA) (#89874) [48]. According to the manufacturer’s protocol, HepG2 cells were harvested and added with reagent A and B into the Eppendorf and vortexed vigorously for 1 h at 4 °C. The reagent C and protein inhibitor were then added into the Eppendorf and centrifuged 700× *g* for 10 min at 4 °C. The pellet was homogenized again following addition of protein inhibitors. Subsequently, the homogenized supernatant was centrifuged at 12,000× *g* for 5 min at 4 °C twice and the supernatant contained mitochondria. Mitochondrial concentration was quantified by BCA assay.

### 4.9. Immunoprecipitation (IP)

Magnetic beads (Bio-Rad Laboratories, Inc., Hercules, CA, USA) were used for IP and to separate specific protein targets [16]. Magnetic beads were incubated with antibody FasL for 10 min at room temperature. Then, the beads–antibody complex was incubated with the target protein Fas. The beads were magnetized using the SureBeads rack and the supernatant was removed. The elution buffer was used for the eluting target protein and analyzed by Western blot analysis.

### 4.10. Protein Extraction and Western Blot Analysis

Proteins from HepG2 cell samples were homogenized in 1 mL RIPA lysis buffer containing a protease inhibitor cocktail (Pierce; Thermo Fisher Scientific, Rockford, IL, USA). Homogenized samples were centrifuged at 4 °C, 12,000 rpm for 10 min. Total protein concentration was quantified by the commercial BCA assay kit (Pierce; Thermo Fisher Scientific, Rockford, IL, USA). Then, 20–30 μg protein extractions were separated by 8% or 10% SDS-poly-acrylamide gels and transferred to nitrocellulose membranes (Whatman, GE Healthcare, Freiburg, Germany). A basic Western blotting procedure was described previously [49]. Primary antibodies against ASK1, Bax, Bcl2, caspase 3, caspase 8, COX-2, COX-4, cytochrome c, Fas, FasL, iNOS, MEK7, and NF-κB were purchased from Santa Cruz (CA, USA). Primary antibodies against Bid and caspase 9 were purchased from Novus Biologicals (Littleton, CO, USA). Primary antibodies against SAPK/JNK and phospho-SAPK/JNK were obtained from Cell Signaling (Beverly, MA, USA). β-actin (A5441) was purchased from Sigma-Aldrich (St Louis, MO, USA) as a loading control. Chemiluminescence detection (Amersham Pharmacia Biotech, Little Chalfont, Bucks, UK) was used to quantify the blots. Quantification of the bands has measured the intensity of the immunoblot band and used ImageQuant™ LAS 4000 mini (GE Healthcare Bio-Sciences AB, Uppsala, Sweden).

### 4.11. Statistical Analysis

Statistical analysis used Sigma Plot 10.0 software (Systat Software, Inc., San Jose, CA, USA) for Windows. The differences between mean values were analyzed by Student’s *t*-test. All experiment results were expressed as mean ± SD. Differences with *p* < 0.05 were considered to be significant.

## 5. Conclusions

In conclusion, the present study suggested that LSE effectively protected from toxic APAP-induced hepatotoxicity by inhibiting apoptosis, suppressing intracellular oxidative stress, and decreasing inflammatory reactions. The study supposed that LSE is a prospective therapeutic potential for overdose APAP antidote.

## Data Availability

Data are available on request to the corresponding author.
